# Nrf2 signaling and autophagy are complementary in protecting lipopolysaccharide/d-galactosamine-induced acute liver injury by licochalcone A

**DOI:** 10.1038/s41419-019-1543-z

**Published:** 2019-04-05

**Authors:** Hongming Lv, Huahong Yang, Zhongfeng Wang, Haihua Feng, Xuming Deng, Genhong Cheng, Xinxin Ci

**Affiliations:** 1 Institute of Translational Medicine, The First Hospital, Jilin University, 130001 Changchun, China; 2 Key Laboratory of Zoonosis, Ministry of Education, Institute of Zoonosis, College of Veterinary Medicine, Jilin University, Changchun, China; 3 Suzhou Institute of Systems Medicine, Suzhou, China; 4 Department of Microbiology, Immunology and Molecular Genetics, University of California, Los Angeles, CA USA

## Abstract

Licochalcone A (Lico A), isolated from Xinjiang licorice Glycyrrhiza inflate, has been shown to have antioxidative potential via the activation of nuclear factor-erythroid 2-related factor 2 (Nrf2) activation, which is involved in the prevention of acetaminophen-induced hepatotoxicity. The purpose of the current study was to further explore the protective effect of Lico A against lipopolysaccharide/d-galactosamine (LPS/GalN)-induced acute liver injury (ALI) and its underlying molecular mechanisms. Our results found that treatment with Lico A significantly reduced in LPS/GalN-induced hepatotoxicity by lessening lethality, alleviating histopathological liver changes, decreasing the alanine transaminase, and aspartate aminotransferase levels, attenuating the secretion of inflammatory cytokines, and regulating oxidative markers. Furthermore, Lico A efficiently alleviated LPS-induced inflammatory response by inhibiting TLR4-MAPK and -NF-κB, as well as the Txnip-NLRP3 signaling pathway. Meanwhile, Lico A induced the activation of Nrf2 and QSTM1 (P62) signaling and promoted autophagy involved in AMP-activated protein kinase (AMPK)-the transcription factor EB (TFEB) signaling, which may contribute to its hepatoprotective activity. Additional mechanistic investigations to evaluate the dependence of the hepatoprotective role of Lico A on Nrf2 revealed that a lack of Nrf2 promoted Lico A-induced autophagy, which contributed to the hepatoprotective effect of Lico A in Nrf2^−/−^ mice. In addition, cotreatment with autophagy inhibitor (3-methyladenine, 3-MA) alleviated but did not abrogate the hepatoprotective effect of Lico A, which may be attributed to its ability to activate Nrf2. Our study firstly suggests that Lico A has protective potential against LPS/GalN-induced hepatotoxicity, which may be strongly associated with activation of Nrf2 and autophagy.

## Introduction

Acute liver injury (ALI) is a dramatic clinical syndrome generally caused by viral infection, toxin, heavy alcohol consumption, and drug poisoning, such as acetaminophen overdose^1^. As we know, ALI induced by acetaminophen overdose predominates in developed western countries, while hepatitis B-induced liver injury mainly occurs in developing countries^2^. So far, it is a dreaded clinical disease that are relevant to a high mortality rate and poor prognosis^3^. Therefore, it is imperative to develop new and effective liver protection agents. Coinjection of mice with lipopolysaccharide and d-galactosamine (LPS/GalN) is a well-established experimental model to investigate the underlying mechanisms of clinical liver disease and to further search for potential therapeutics^4^. LPS, the main inflammatory bacterial component, is usually used to trigger immune cell activation and initiation of the inflammation response. GalN, the liver toxin, is sensitized to LPS lethality by inhibiting protein and RNA synthesis^5^. When macrophages are attacked by LPS, the production of proinflammation cytokines and reactive oxygen species (ROS) were released, which can augment liver damage following exposure to the hepatotoxicant LPS^6^. Therefore, inhibiting inflammation and/or oxidative stress may be a potential preventive measure for the development of acute liver injury.

Previous reports have shown that LPS/GalN-induced acute liver injury mainly involves the release of massive inflammatory cytokines, such as interleukin (IL)-1β, IL-6, and tumor necrosis factor (TNF)-α. Toll-like receptors (TLRs) are a class of pattern recognition receptors (PRRs) that interact with components of pathogens and mediate the intracellular inflammatory responses^7^. When LPS attacked hepatocytes, TLR4 can be recognized by LPS, and can then further activate the nuclear transcription NF-κB and mitogen-activated protein kinase (MAPK), which include the c-Jun NH2-terminal kinase (JNK), extracellular signal-regulated kinase (ERK) and p38^8,9^. NF-κB and the MAPK pathway regulate inflammatory cytokines release, and are considered the main signaling pathways related to the acute inflammatory response. Inflammation is often accompanied by formation of reactive oxygen species (ROS) and oxidative stress. In addition, oxidative stress also accelerates the inflammatory response by activating pro-inflammatory pathways, including the well-known NOD-like receptor protein 3 (NLRP3) inflammasome pathways. Thioredoxin-interacting protein (Txnip) has been shown to be a key signaling molecule that links oxidative stress to inflammasome activation^10^. Activation of the NLRP3 inflammasome via Txnip-NLRP3 interaction contributes to the LPS/GalN-induced inflammatory responses, which may be associative with the development of fulminant hepatic injury^11^. Given that HO-1 overexpression may protect the liver against LPS/GalN-induced inflammation by inhibiting the NLRP3 signaling pathway^11^, the search for potential antioxidative pathways that can be regulated by endogenous or exogenous compounds may become prospective therapeutic targets for human diseases.

Among these antioxidative pathways, nuclear factor erythroid 2-related factor 2 (Nrf2), is a key transcription factor that is required to ameliorate various oxidative stress- and inflammation-associated diseases. Under basal conditions, Nrf2 is constitutively kept in the cytoplasm and degraded via binding to its main antagonist, Kelch-like ECH-associated protein 1 (Keap1). Upon oxidative stress, Nrf2 dissociates from Keap1 and translocates into the nucleus and regulates the expression of various antioxidant genes, including heme oxygenase-1 (HO-1)^12^. The induction of Nrf2 not only rescues the organisms from oxidative injury but also exerts a protective effect against inflammation in the pathogenesis of liver injury both in vitro and in vivo^13,14^. Importantly, autophagy is a homeostatic degradative process that removes damaged organelles or turns over cytoplasmic constituents via lysosomal compartments in eukaryotic cells^15^. Reports in recent years have shown that autophagy is widely considered a crucial modulator of cell survival and homeostasis, while deficiency in autophagy promotes inflammatory responses and oxidative stress and ultimately causing various pathological diseases in different tissues^16,17^. In particular, recent observations have shown an inverse relationship between autophagy induction and maturation of NLRP3 inflammasomes in macrophages^18^. Therefore, autophagy may reduce proinflammatory cytokines and alleviate acute liver injury via inhibiting the activation of the NLRP3 inflammasome in macrophages and in mice^19^. In addition, activation of autophagy by pharmacotherapy can protect against acute liver injury induced by various stimulus^20^. Thus, strategies designed to resolve the dysfunction of autophagy seem to be beneficial for the treatment of acute liver injury.

Licochalcone A (Lico A) is a natural flavonoid compound that isolated from the root of the Xinjiang licorice Glycyrrhiza inflate. Recent studies have revealed that Lico A possesses various biological activities, including anti-inflammatory, antioxidant, and antimicrobial activities^21,22^. Furthermore, Lico A exhibits anti-cancer properties via activating autophagy^23^. Researchers have reported that Lico A showed hepatoprotective effects in liver damage induced by acetaminophen and carbon tetrachloride^24,25^. However, the protective effect of Lico A against LPS/GalN-induced liver injury has not been reported. The aim of this study is to investigate the protective effects of Lico A on LPS/GalN-induced liver injury and potential mechanisms, and whether it contributes to the activation of Nrf2 and the autophagy signaling pathway.

## Results

### Lico A treatment protected mice from acute liver injury (ALI) caused by LPS/GalN administration

**Fig. 1:**
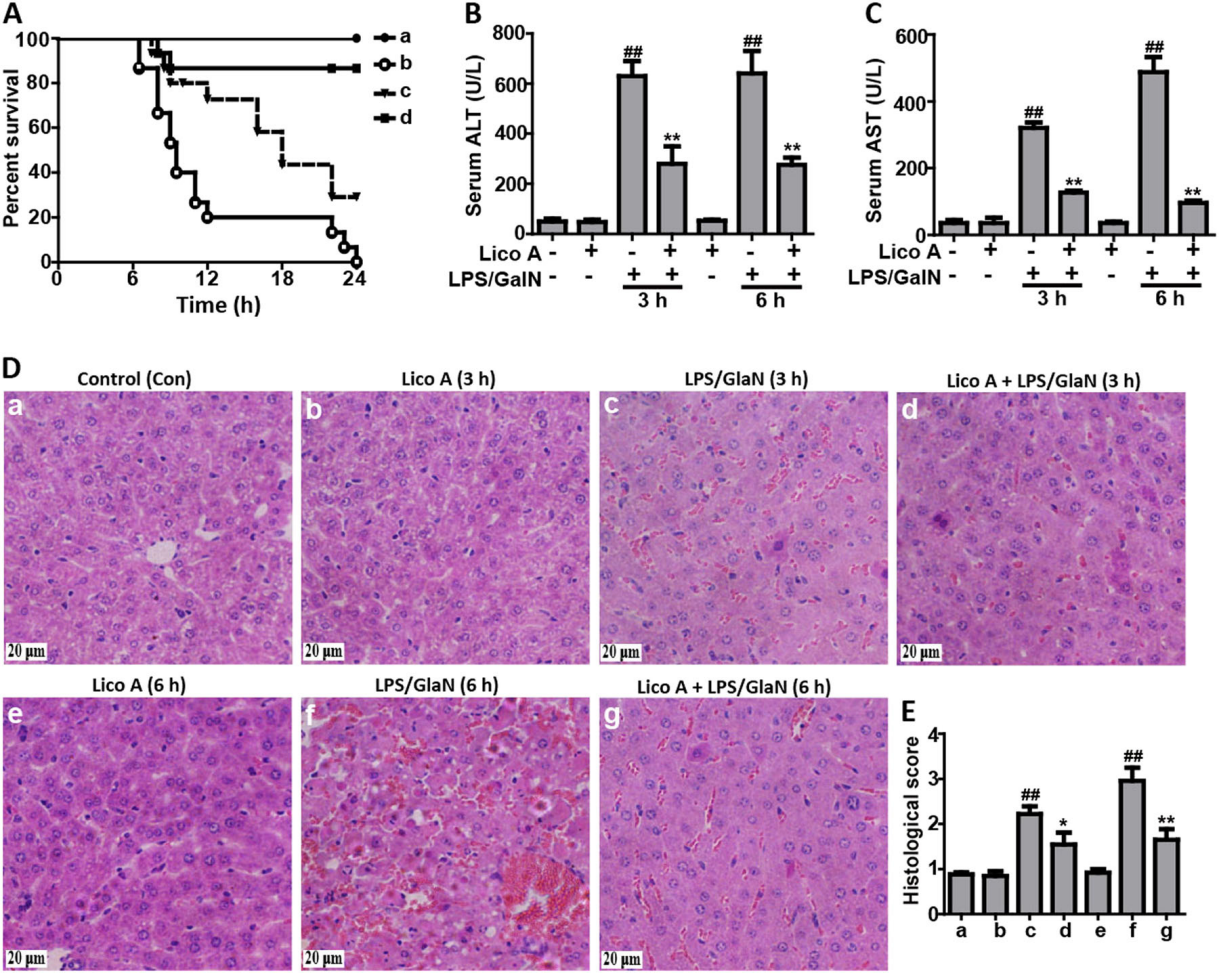
The protective effects of Lico A-treated on LPS/GalN-induced ALI. Lico A (50 or 100 mg/kg) was administered intraperitoneally to mice for twice (interval for 12 h), followed by exposed to LPS (30 μg/kg) and D-GalN (600 mg/kg), which is abbreviated as LPS/GalN. **a** Survival rate of mice was observed within 24 h after LPS/GalN administration. (a) Control and Lico A (100 mg/kg) group; (b) LPS/GalN group; (c) Lico A (50 mg/kg)+LPS/GalN; (d) Lico A (100 mg/kg)+LPS/GalN. **b**, **c** Injection with LPS/GalN for 3 h and 6 h, serum of mice were collected for assessment of ALT and AST levels. **d** Livers (*n* = 5) from each experimental group were subjected to stain with hematoxylin and eosin (**h** and **e**)-stained (magnification ×400). **e** The stained sections were defined as using a four-point scale from 1 to 4, with 1, 2, 3, and 4, which represents (1) no damage, (2) mild damage, (3) moderate damage, and (4) severe damage, respectively. Similar results were obtained from three independent experiments. All data are presented as means±SEM (*n* = 5 in each group). ^*^p < 0.05 and ^**^p < 0.01 vs. Control group; ^##^*p* < 0.01 vs LPS/GalN group

To investigate whether Lico A could alleviate LPS/GalN-caused liver injury in mice, survival rate analysis firstly was evaluated within 24 h after GalN/LPS injection. In this study, our results showed that the mice in the LPS/GalN group began to perish 7 h after LPS/GalN challenge and that the survival rate of mice dropped to 0%, whereas after Lico A treatment it was enhanced to ~90% (100 mg/kg) or 30% (50 mg/kg), respectively (Fig. 1a). ALT and AST levels in serum are acknowledged as key markers of hepatic injury, therefore the levels in the serum of mice with ALI were detected. As presented in the Fig. 1b, c, Lico A treatment effectively reduced ALT and AST levels in serum that was increased by LPS/GalN compared to the control group. Meanwhile, histological change of mice liver in the LPS/GalN group displayed a noticeably disturbed architecture, such as hemorrhage, neutrophil infiltration, and hepatocyte necrosis whereas these liver alterations were efficiently relieved by Lico A treatment. These results were presented as liver injury score (Fig. 1d, e).

### Lico A treatment inhibited LPS/GalN-induced TNF-α, IL-6, and IL-1β secretion in mice

**Fig. 2:**
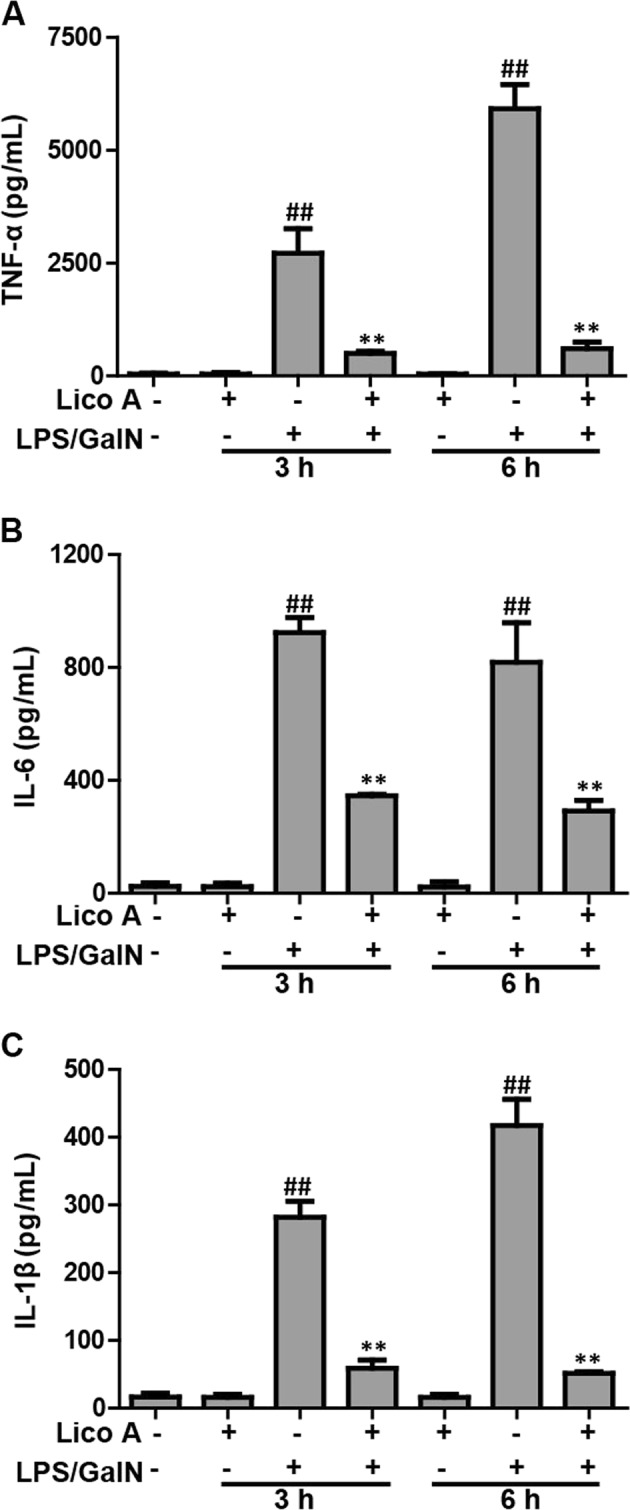
Effect of Lico A-treated on the secretion of TNF-α, IL-6, and IL-1β in mice with LPS/GalN-induced ALI. Mice at 3 h or 6 h after LPS/GalN injection, serum of mice were collected for assessment of production of inflammatory cytokines. **a**–**c** Effects of Lico A on LPS/GalN-induced serum TNF-α, IL-6, and IL-1β generation. Similar results were obtained from three independent experiments. All data are presented as means±SEM (*n* = 5/group). ^**^p < 0.01 vs. Control group; ^##^*p* < 0.01 vs. LPS/GalN group

Given levels of TNF-α, IL-6, and IL-1β are relevant to liver injury, the inflammatory cytokine levels were measured in serum of mice that were LPS/GalN-induced using ELISA. As presented in Fig. 2, LPS/GalN remarkably stimulated the secretion of IL-6, IL-1β, and TNF-α in serum compared to the control group, whereas Lico A treatment lessened the inflammatory cytokine production as a result of LPS/GalN administration, suggesting that Lico A possessed effective anti-inflammatory activity.

### Lico A treatment alleviated LPS/GalN-triggered oxidative insult in mice with ALI

**Fig. 3:**
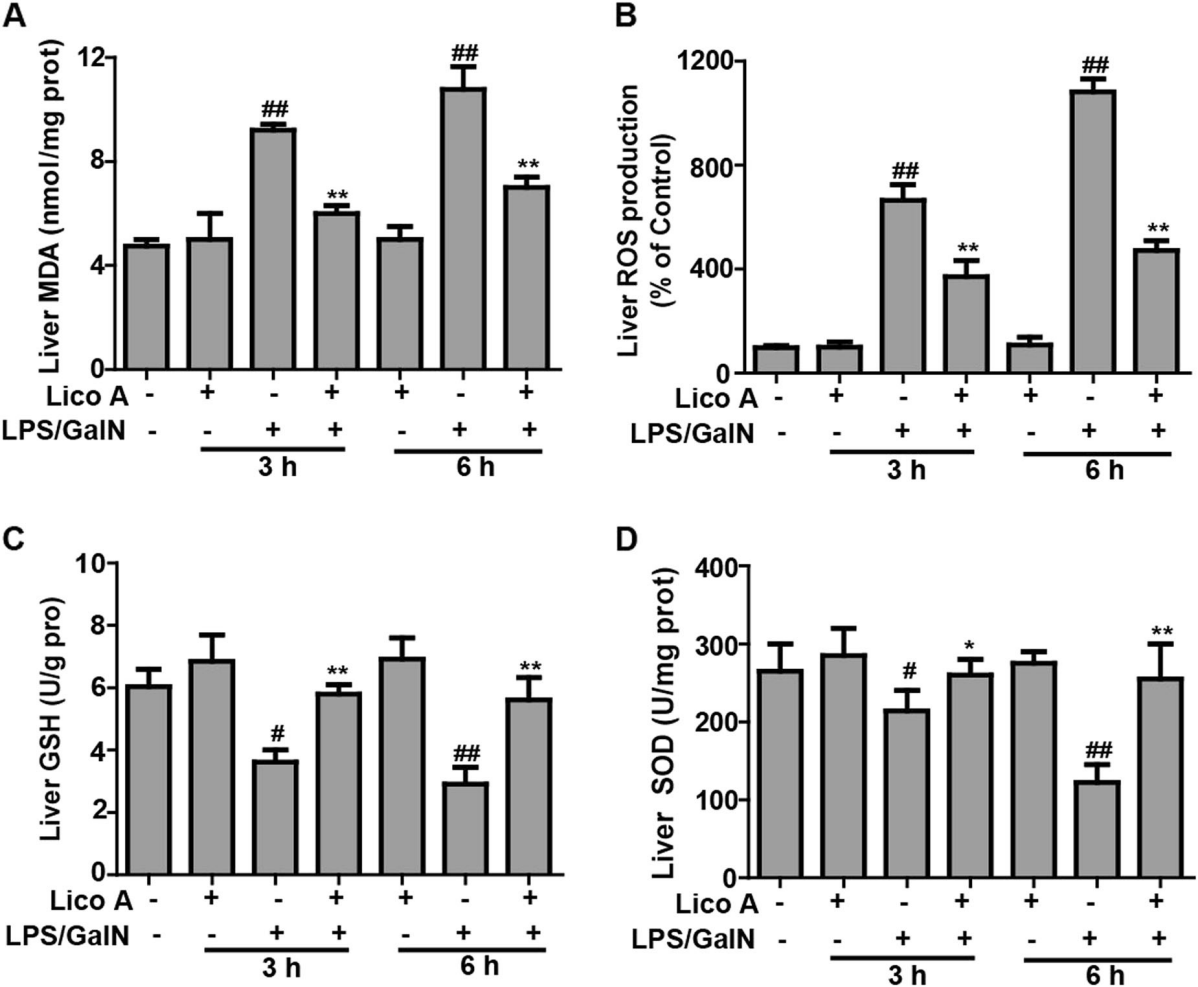
Effects of Lico A-treated on levels of oxidative markers in LPS/GalN -induced ALF. Mice at 3 h or 6 h after LPS/GalN injection, liver tissues of mice were collected for assessment of generation of ROS and MDA, depletion of SOD and GSH. **a**–**d** Effect of Lico A on the levels of ROS, MDA, SOD, and GSH. Similar results were obtained from three independent experiments. All data are presented as means±SEM (*n* = 5 in each group). ^*^p < 0.05 and ^**^p < 0.01 vs. Control group; ^#^*p* < 0.05 and ^##^*p* < 0.01 vs LPS/GalN group

Oxidative insult is also a major problem that is caused by liver injury induced by LPS/GalN. Therefore, we measured whether Lico A treatment can improve LPS/GalN-triggered oxidative damage. In the present study, LPS/GalN significantly increased the excessive accumulation of MDA and ROS, and led to the depletion of GSH and SOD, resulting in oxidative insult in the liver of mice. However, Lico A treatment efficiently reversed these effects caused by LPS/GalN (Fig. 3), indicating that Lico A treatment relieved hepatic injury by suppressing oxidative stress stimulated by LPS/GalN.

### Lico A treatment inhibited LPS/GalN-activated TLR4-NF-κB and -MAPK signaling pathway in mice with ALI

**Fig. 4:**
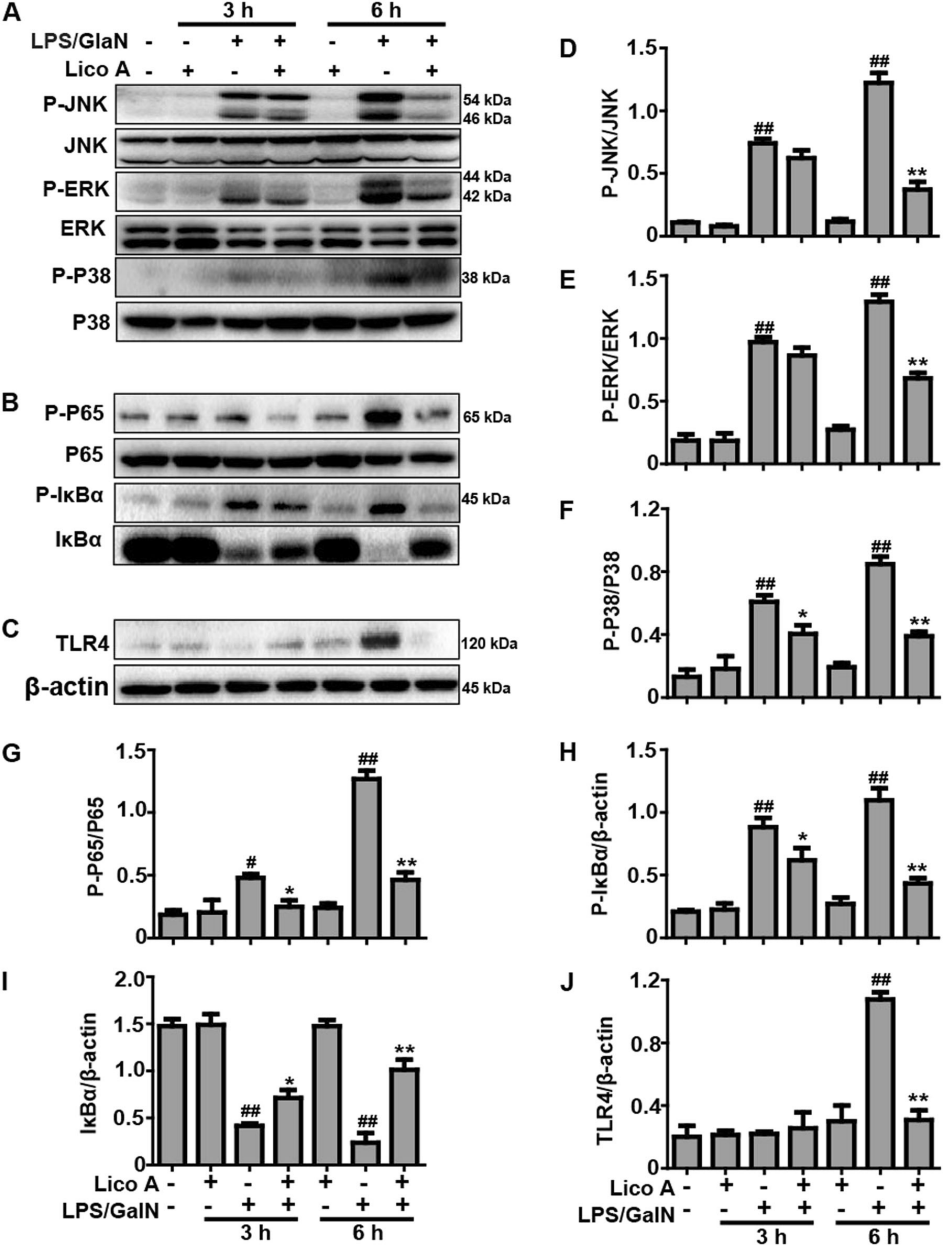
Effects of Lico A-treated on LPS/GalN-activated TLR-MAPK and -NF-κB signaling pathway in ALI. Liver tissues were collected from the mice 3 h and 6 h after LPS/GalN challenge and analyzed by western blot. **a** Effects of Lico A on P-JNK, P-ERK, and P-p38 protein expression were measured by western blotting analysis. **b** Effects of Lico A on P-IκBα, IκBα, P-NF-κB (p65) and NF-κB (p65) protein expression were measured by western blot. **c**, **a** Effects of Lico A on the TLR4 protein expression were measured by western blot. **d**–**j** Quantification of relative protein expression was performed by densitometric analysis. Similar results were obtained from three independent experiments. All data are presented as means±SEM (*n* = 5/group). ^*^p < 0.05 and ^**^p < 0.01 vs. Control group; ^#^*p* < 0.05 and ^##^*p* < 0.01 vs LPS/GalN group

The TLR4 signaling pathway acts, upstream of NF-κB and MAPK, and has been recognized as being a key signaling pathway mediating inflammatory responses that play a vital role in various inflammation-associated diseases, including LPS/GalN-induced ALI. The following experiments were performed to determine the effects of Lico A treatment on LPS/GalN-activated TLR-NF-κB and -MAPK signaling pathway. As shown in Fig. 4, compared to the LPS/GalN-challenged group, Lico A treatment significantly inhibited the phosphorylation of JNK, ERK, P38, and NF-κB (P65), blocked the phosphorylation and degradation of IκBα, and reduced the expression of the TLR4 protein at LPS/GalN-exposed mice at 6 h, suggesting that inflammation inhibited by Lico A may partly attribute to suppress the activation of TLR4-NF-κB and -MAPK signaling pathway.

### Lico A treatment suppressed LPS/GalN-activated Txnip-NLRP3 inflammasome signaling pathway in mice with ALI

**Fig. 5:**
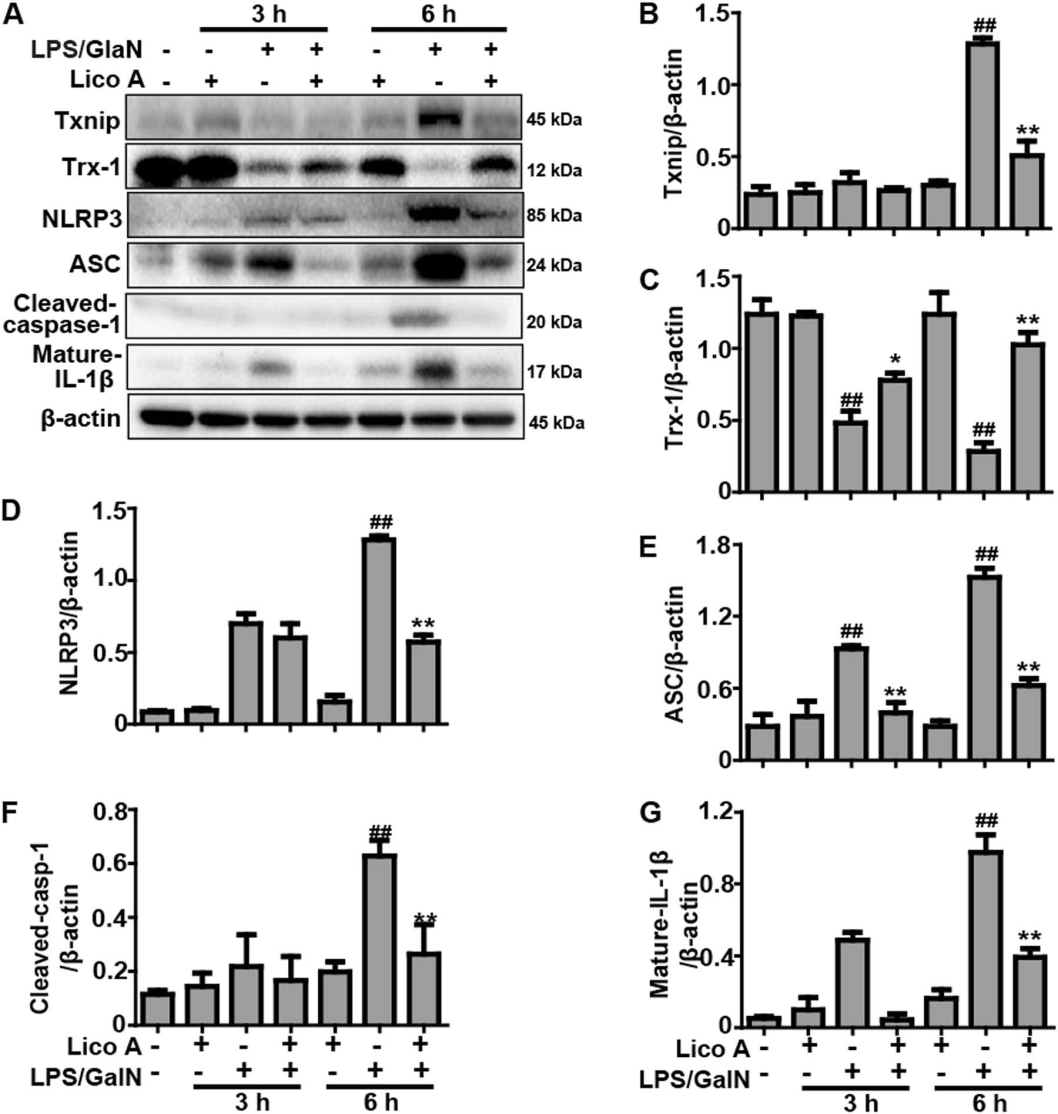
Effects of Lico A-treated on LPS/GalN-induced Txnip-NLRP3 inflammasome signaling pathway in ALI mice. Liver tissues were collected from the mice 3 h and 6 h after LPS/GalN challenge and analyzed by western blot. **a** Effects of Lico A treatment on LPS/GalN-induced Txinp, Trx-1, NLRP3, ASC, Cleaved-caspase-1, and Mature-IL-1β were measured by western blotting. **b**–**g** Quantification of relative protein expression was performed by densitometric analysis and β-actin was used as an internal control. Similar results were obtained from three independent experiments. All data are presented as means±SEM (*n* = 5/group). ^*^p < 0.05, ^**^p < 0.01 vs. LPS/GalN group; ^##^*p* < 0.01 vs. Control group

Previous reports revealed that a GalN/LPS-activated inflammation reaction is regulated by Txnip-NLRP3 inflammsome activation, which is interrelated to the occurrence and development of liver injury. Western blot analysis demonstrated that expression of Txinp, NLRP3, ASC, Cleaved-caspase-1 (p20), and Mature-IL-1β (p17) protein were elevated, and that the expression of the Trx-1 protein was decreased 6 h after LPS/GalN injection, whereas the protein expression was only slightly changed 3 h after LPS/GalN injection. Interestingly, Lico A treatment not only dramatically suppressed the expressions of Txnip, NLRP3, ASC, Cleaved-caspase-1, and Mature-IL-1β protein (Fig. 5), but also enhanced the expression Trx-1 protein in LPS/GalN-induced ALI, implying that inflammation inhibited by Lico A may also be partly responsible for suppressing activation of the Txnip-NLRP3 inflammasome.

### Lico A treatment upregulated the P62-Nrf2/HO-1 signaling pathway in mice with LPS/GalN-induced ALI

**Fig. 6:**
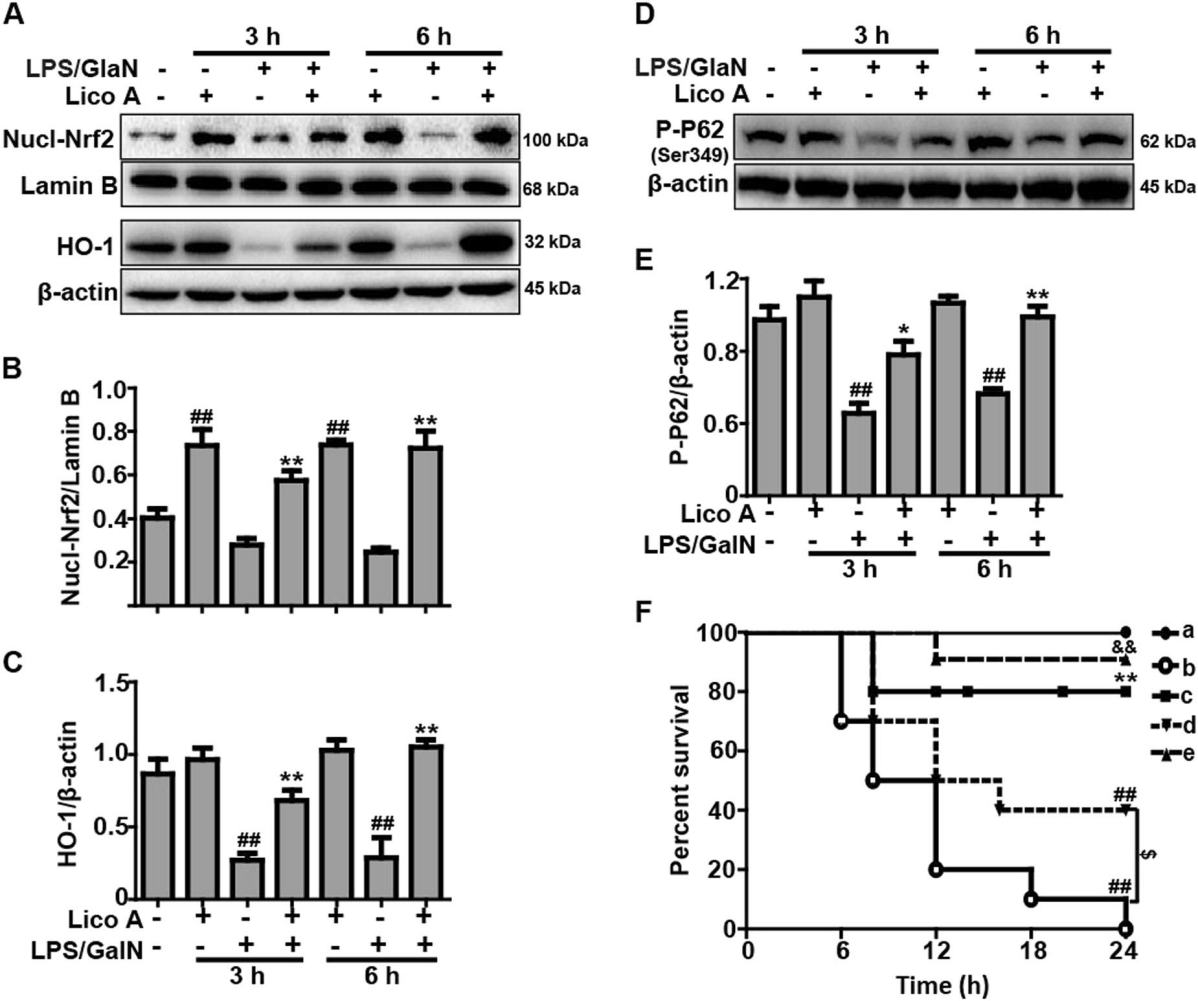
Effects of Lico A-treated on the upregulation of P62-Nrf2/HO-1 pathway in LPS/GalN-induced ALI. **a** Liver tissues were collected from the mice 3 h and 6 h after LPS/GalN challenge and analyzed by western blot for the assessment of the nuclear levels of Nrf2, and HO-1 protein expression. Moreover, **d** the effect of Lico A on the phosphorylation of P62 at ser349. **b**, **c**, **e** Quantification of relative protein expression was performed by densitometric analysis. Lamin B and β-actin were used as an internal control. WT and Nrf2^−/−^ mice were intraperitoneally injected Lico A (100 mg/kg) with mice for twice at a 12-h (interval for 12 h), followed by subjected to LPS/GalN. **f** The survival rates of the mice (*n* = 10/group) were observed within 24 h after LPS (30 μg/kg) and GalN (600 mg/kg) exposure. (a) WT/KO Control and Lico A group; (b) WT LPS/GalN group; (c) WT Lico A+LPS/GalN group; (d) KO LPS/GalN group; (e) KO Lico A+LPS/GalN group. Similar results were obtained from three independent experiments. All data are presented as means±SEM (*n* = 5/group). ^##^*p* < 0.01 vs. WT control group; ^*^p < 0.05, ^**^p < 0.01 vs. WT LPS/GalN group; ^$$^*p* < 0.01 vs. KO control group; ^&^*p* < 0.01 vs. KO LPS/GalN group

Previous reports have revealed that the Nrf2-mediated signaling pathway inhibits inflammation and oxidative stress which can attenuate hepatic injury resulting from a variety of factors, such as LPS/GalN. The findings of the current study indicated that the Lico A treatment efficiently facilitated the nuclear transcription of Nrf2 and the expression of HO-1 protein when compared with the LPS/GalN-treated group (Fig. 6a–c). Furthermore, recent report indicated that the phosphorylation of P62 plays a vital role in inducing the nuclear transcription of Nrf2. Thus, we determined whether Lico A could increase the phosphorylation of P62. The results showed that Lico A treatment dramatically promoted the phosphorylation of P62 at ser349 in LPS/GalN-induced ALI (Fig. 6d, e). Subsequently, in survival rate analysis, the final survival rate was 0% (control) versus 80% (Lico A-treated group) in WT mice, whereas it was 40% (control) versus 90% (Lico A-treated group) in Nrf2^−/−^ mice, suggesting that Nrf2^−/−^ mice were less susceptible to LPS/GalN and developed less severe liver damage compared to WT mice (Fig. 6f). Here, we speculate that Lico A may mediate others signaling to equally or predominantly play a protective role in LPS/GalN-induced ALI.

### Lico A-induced autophagy activation is strengthened by Nrf2 deficiency in mice induced by LPS/GalN

**Fig. 7:**
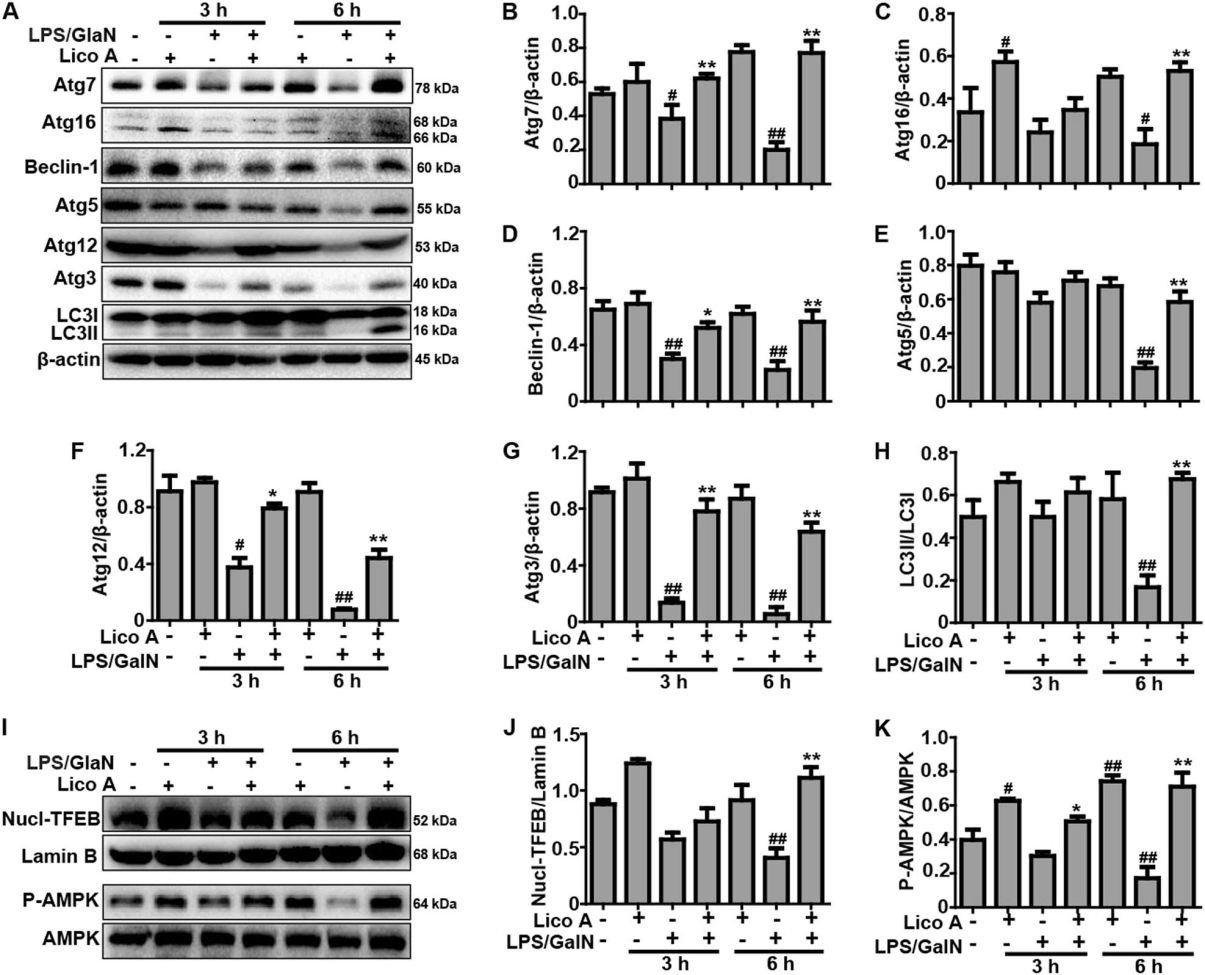
The improvement effect of Lico A-mediated autophagy on liver injury induced by LPS/GalN in mice. Liver tissues were collected from the mice 3 h and 6 h after LPS/GalN challenge and analyzed by western blot. **a** Effects of Lico A on the protein expression of Atg7, Atg16, Beclin-1, Atg5, Atg12, Atg3, and LC3 conservation were measured by western blotting. Moreover, anti-Atg5 and anti-Atg12 antibodies detect the identical bands corresponding to Atg12-Atg5 conjugates. Additionally, **i** the effect of Lico A on the phosphorylation of AMPK and the nuclear translocation of TFEB. **b**–**h**, **j**, **k** Quantification of relative protein expression was performed by densitometric analysis. β-actin was used as an internal control. Similar results were obtained from three independent experiments. All data are presented as means±SEM (*n* = 5/group). ^*^p < 0.05, ^**^p < 0.01 vs. LPS/GalN group; ^#^*p* < 0.05, ^##^*p* < 0.01 vs. Control group

**Fig. 8:**
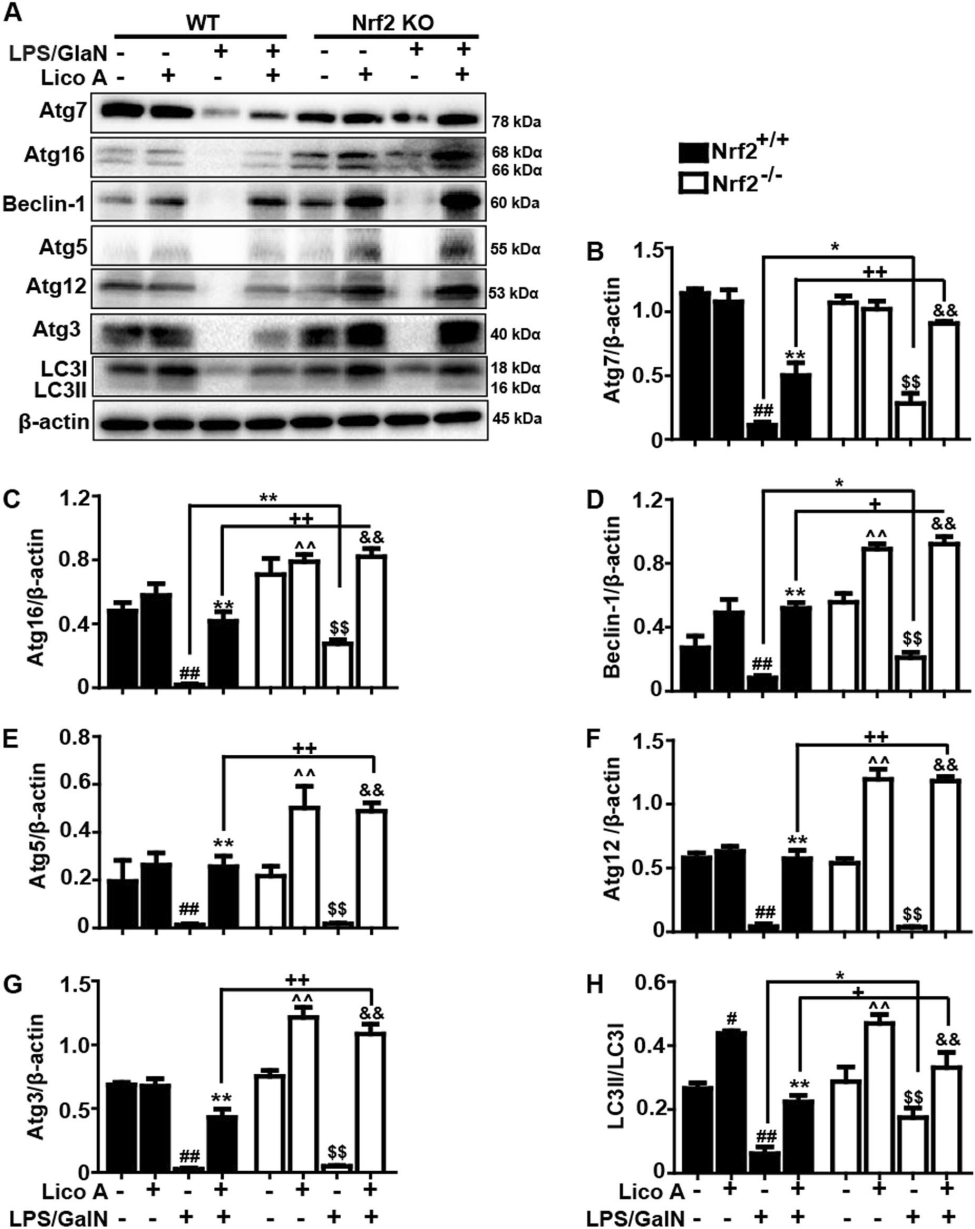
Nrf2 deficiency strengthened autophagy caused by Lico A in mice with LPS/GalN-induced ALI. WT and Nrf2^−/−^ mice were intraperitoneally injected Lico A (100 mg/kg) with mice for twice at a 12-h (interval for 12 h), followed by subjected to LPS/GalN (30 μg/kg; 600 mg/kg). Liver tissues were collected from the mice 6 h after LPS/GalN challenge and analyzed by western blotting. **a** Effects of Lico A on the protein expression of Atg7, Atg16, Beclin-1, Atg5, Atg12, Atg3, and LC3 conservation were measured by western blot. Moreover, anti-Atg5 and anti-Atg12 antibodies detect the identical bands corresponding to Atg12-Atg5 conjugates. **b**–**h** Quantification of relative protein expression was performed by densitometric analysis. β-actin was used as an internal control. Similar results were obtained from three independent experiments. All data are presented as means±SEM (*n* = 5 in each group). ^##^*p* < 0.01 vs. WT Control group; ^*^p < 0.05, ^**^p < 0.01 vs. WT LPS/GalN group; ^^^^*p* < 0.01 vs. WT Lico A group; ^+^*p* < 0.05, ^++^*p* < 0.01 vs. WT LPS/GalN+Lico A group; ^$$^*p* < 0.01 vs. KO Control group; ^&^*p* < 0.05, ^&&^*p* < 0.01 vs. KO LPS/GalN group

Considering that enhanced autophagy negatively modulates the inflammatory response and oxidative stress, and plays an essential role in the amelioration of hepatotoxicity induced by LPS/GalN, further indicated that the importance measuring protein levels of autophagy genes including Atg7, Atg12, Atg16, Beclin-1, Atg5, Atg3, and LC3II conversion by western blot. The data in this study showed that the protein expression of these autophagy genes was declined by LPS/GalN exposure for 3 h and 6 h, and the protein expression was significantly recovered by Lico A treatment (Fig. 7a-h). Meanwhile, to further investigate the underlying mechanisms of LicoA-induced autophagy, we measured whether it could result in the phosphorylation of AMPK and the nuclear translocation of TFEB. As present in Fig. 7i–k, Lico A obviously enhanced the AMPK phosphorylation and the TFEB nuclear translocation. Furthermore, to explore a possible association between Lico A-mediated autophagy activation and Nrf2 upregulation, WT mice and Nrf2^−/−^ mice were constructed in order to analyze the protein levels of Atg7, Atg12, Atg16, Beclin-1, Atg5, Atg3, and LC3II conversion. As illustrated in Fig. 8, we surprisingly discovered that LPS/GalN reduced less Atg7, Atg16, Beclin-1, and LC3 activation in Nrf2^−/−^ mice compared to WT mice, and that Lico A recovered the autophagy protein expression levels that reduced by LPS/GalN in WT mice was found to be significantly strengthened in Nrf2^−/−^ mice. These investigations indicated that Nrf2 deficiency may promote compensatory effects of autophagy activation to enhance the protective ability against LPS/GalN-induced ALI in mice.

### Lico A improved ALI caused by LPS/GalN dependent upon autophagy activation in mice

**Fig. 9:**
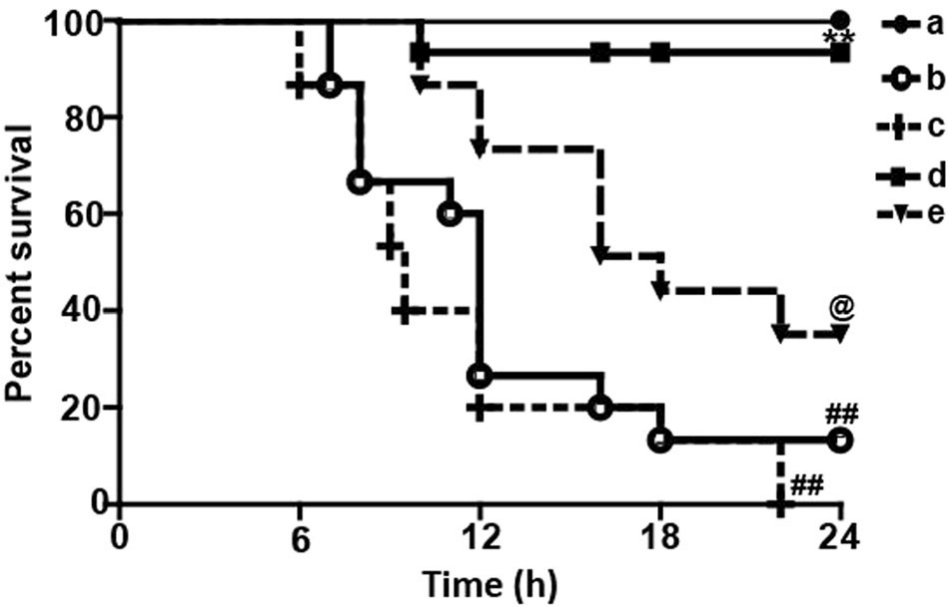
The attenuation effect of Lico A-mediated autophagy on liver injury induced by LPS/GalN in mice. WT mice were intraperitoneally injected 3-MA (20 mg/kg) for 2 h, and then Lico A (100 mg/kg) with mice for twice at a 12-h (interval for 12 h), followed by subjected to LPS (10 μg/kg) /GalN (700 mg/kg). The survival rates of the mice (*n* = 10 / group) were observed within 24 h after LPS (10 μg/kg) and GalN (700 mg/kg) exposure. **a** Control and Lico A group; **b** LPS/GalN group; **c** 3-MA+LPS/GalN; **d** Lico A+LPS/GalN; **e** 3-MA+Lico A+LPS/GalN. Similar results were obtained from three independent experiments. All data are presented as means±SEM. ^##^*p* < 0.01 vs. Control group; ^**^p < 0.01 vs. LPS/GalN group; ^@^*p* < 0.01 vs. LPS/GalN+3-MA group

To further verify the contribution of autophagy and benefits of Lico A treatment in acute liver injury, mice were pretreated with 3-methyladenine (3-MA) before treatment with LPS/GalN. The survival rate of mice in the LPS/GalN (10 μg/kg/700 mg/kg) group was ~15%, whereas the survival rate after 3-MA treatment was 0%. Nevertheless, the survival rate in mice cotreated with 3-MA and Lico A was close to 40% (Fig. 9). Accordingly, based on Figs. 6F and 9, we speculated that Nrf2 deficiency enhanced autophagy as a results of Lico A by improving its ability to resist LPS/GalN-induced hepatotoxicity, thereby explaining why Nrf2^−/−^ mice have the higher survival rate than WT mice.

## Discussion

Acute liver injury (ALI) is a fatal disease associated with many complications^26^. Lethal doses of LPS/GalN in mice have become one of the most popular experimental models for screening potential therapeutic agents for ALI^4^. LPS/GalN-induced liver injury largely depends on the LPS-induced pro-inflammatory cytokines and reactive oxygen species (ROS)^27^. Accordingly, any approach that relieves inflammatory responses and oxidative stress can be used to prevent or treat ALI. Lico A, a natural flavonoid compound, has been reported to have a potential against APAP-induced hepatotoxicity which may be strongly related to the Nrf2-mediated defense mechanism^24^. In the current study, we tested the hepatoprotective effect of Lico A on LPS/GalN-induced acute liver injury and found for the first time that Lico A possesses a potent anti-inflammatory effect against LPS/GalN-induced hepatotoxicity through mechanisms relying on Nrf2 activation and autophagy induction.

There is growing evidence that acute liver injury (ALI) resulting from LPS/GalN is characterized by high mortality, elevated ALT and AST in the serum and a pathological change in the liver^28^. As previously reported, we found that LPS/GalN significantly increased mortality, serum ALT and AST levels, and liver histopathological changes. However, Lico A treatment significantly prevented these elevations, suggesting that Lico A protected liver tissues against the toxic effects of LPS/GalN. Emerging evidence has suggested that LPS/GalN gives rise to hepatic injuries by inducing oxidative stress and inflammatory responses^13,29,30^. We further evaluated the effect of Lico A treatment on inflammatory cytokines in serum and oxidative markers in the liver. Our results showed that Lico A significantly reduced TNF-α, IL-6 and IL-1β generation in the serum, alleviated MDA and ROS levels in the liver, and reversed GSH and SOD depletion in liver, indicating that Lico A treatment dramatically alleviated the inflammatory response and oxidative injury in mice.

The signal cascades that generate proinflammatory cytokines are mainly regulated by NF-κB and MAPK mediated signaling^31^. TLR4 recognizes extracellular LPS and results in activation of TLR4-MyD88 followed by activation of NF-κB by phosphorylation and degradation of IκBα, as well as p38, ERK and JNK phosphorylation, which produces inflammatory factors and ultimately causes liver inflammation^32,33^. It has been reported that Lico A inhibits LPS-induced inflammatory response through the NF-κB and p38/ERK MAPK signaling pathway in vitro and in vivo^34^. Our results showed that Lico A inhibited LPS/GalN-induced phosphorylation of p38, ERK, JNK, NF-κB (p65), and IκBα, and blocked LPS/GalN-induced TLR4 activation and IκBα degradation in liver tissues, specifically 6 h after LPS/GalN challenge. Furthermore, the activation of NF-κB is an essential initial step for the priming of NLRP3 activation, and ROS generated from NF-κB-mediated inflammation also serves as a danger signal that activates NLRP3^35^. Once NLRP3 is activated, the recruitment of ASC begins, followed by activation of caspase-1, and the maturation of inflammatory cytokines IL-1β, which is associated with the pathogenesis of liver injury^36^. Our results indicated that Lico A dramatically inhibited LPS/GalN-induced NLRP3, ASC, cleave-caspase-1, and mature-IL-1β protein expression. Recent reports have demonstrated that excessive ROS accumulation resulting from cellular stress leads to the separation of thioredoxin-interacting protein (Txnip) from thioredoxin-1 (Trx-1), and the subsequent binding of Txinp to NLRP3, which then triggers NLRP3 inflammasome activation^37^. In addition, activation of the NLRP3 inflammasome was caused by a LPS/GalN-induced inflammatory response through Txnip-NLRP3 interaction^11^. In this study, additional results revealed that Lico A dramatically inhibited LPS/GalN-induced expression of the Txnip protein and a concomitant decrease of Trx-1 protein expression, which has been proved to be a key signaling molecule, linking oxidative stress to inflammasome activation. In this process, HO-1 overexpression could restrain their interaction to attenuate the liver injury induced by LPS/GalN^37^. Based on the above results, we hypothesized that the ability of Lico A to alleviate inflammatory responses and liver injury might be involved in the ability to counterbalance oxidative stress.

In fact, Nrf2 is a key player in the cellular antioxidant defense system, and its protective role in LPS/GalN-induced hepatotoxicity is well established^38^. Our pervious experiments have shown that the upregulation of Nrf2 blocked Txnip-NLRP3 inflammasome activation and enhanced the induction of HO-1, which improved LPS-induced acute lung injury^39^. Moreover, it is reported that the mechanisms of Nrf2 activation is associative with P62-Nrf2-Keap1 axis^40^, and Lico A could upregulate Nrf2 signaling through the phosphorylation of P62 at serine 349 in the arthritis model of mice^41^. In the study, we also explored the effect of Lico A on examine the phosphorylation of P62. The findings suggested that Lico A effectively induced Nrf2 translocation, P62 ^Ser349^ phosphorylation, and HO-1 expression in mice with LPS/GalN-induced liver injury. These results indicated that Nrf2 played an essential role in protecting against LPS/GalN-induced tissues damage. Based on the results above, we performed further mechanistic investigations to evaluate whether the hepatoprotective effect of Lico A was dependent on Nrf2 by using Nrf2 knockout mice. In the survival rate analysis, the final survival rate was 0% (LPS/GalN group) versus 80% (Lico A-treated+LPS/GalN group) in WT mice, whereas it was 40% (LPS/GalN group) versus 90% (Lico A-treated+LPS/GalN group) Nrf2^−/−^ mice, suggesting that Nrf2^−/−^ mice were less susceptible to LPS/GalN and developed less severe liver damage compared to WT mice. Here, we speculated that Lico A may mediate other signaling to equally or predominantly play a protective role in LPS/GalN-induced ALI. Until now, substantial evidence has revealed that enhanced autophagy negatively modulates inflammatory response, exhibiting an essential role in the amelioration of hepatotoxicity induced by LPS/GalN^42^. Moreover, several reports have maintained that sustained autophagy could relieve acetaminophen-induced liver injury by inhibiting oxidative stress^43,44^. Our next results showed that Lico A treatment induced autophagy activation via promoting protein levels of Atg7, Atg5, Beclin-1, Atg5, Atg3, and LC3II conversion that were reduced by LPS/GalN. Importantly, the transcription factor EB (TFEB) is recognized to act as linking autophagy to lysosomal biogenesis and its activity is modulated by mTOR and AMPK signaling pathways^45–47^. Beyond that, Xue et al noted that Lico A could promote autophagy via the inhibition of PI3K/Akt/mTOR signaling pathway activation in breast cancer cells^48^. Therefore, it is necessary to explore the effect of Lico A on TFEB, mTOR, and AMPK signaling pathway. In the current study, we found that Lico A could enhance the nuclear transcription of TFEB and the phosphorylation of AMPK, but it could not exhibit the induction of mTOR in LPS/GlaN-induced ALI, indicating that Lico A-induced autophagy activation may be dependent on the AMPK-TFEB signaling pathway.

Interestingly, in the study of the relationship between Nrf2 and autophagy activation, we uncovered that LPS/GalN reduced less Atg7, Atg16, Beclin-1, and LC3 activation in Nrf2^−/−^ mice compared to WT mice, and Lico A induced more these autophagy protein expressions in Nrf2^−/−^ mice than WT mice, suggesting that a knockout of Nrf2 may promote compensatory effects of autophagy activation, which may explain the above survival data and also contributes to the hepatoprotective effect of Lico A in Nrf2^−/−^ mice. To further confirm the contribution of autophagy and benefits of Lico A treatment in acute liver injury, mice were pretreated with 3-methyladenine (3-MA) before treatment with LPS/GalN. These observations unveiled that the 3-MA treatment exacerbated liver damage characterized by increased mortality in response to the LPS/GalN challenge. Furthermore, co-treatment with the 3-MA and Lico A increased the mortality rate compared with the Lico A-treated group in LPS/GalN-induced ALI, suggesting that inhibition of autophagy by 3-MA partially weakened the Lico A mediated protective effect on LPS/GalN in mice. Taken together, we hypothesize that both Lico A-mediated autophagy activation and Nrf2 upregulation ameliorates LPS/GalN-caused liver injury, while lack of Nrf2 may enhance compensatory effects of autophagy activation.

**Fig. 10:**
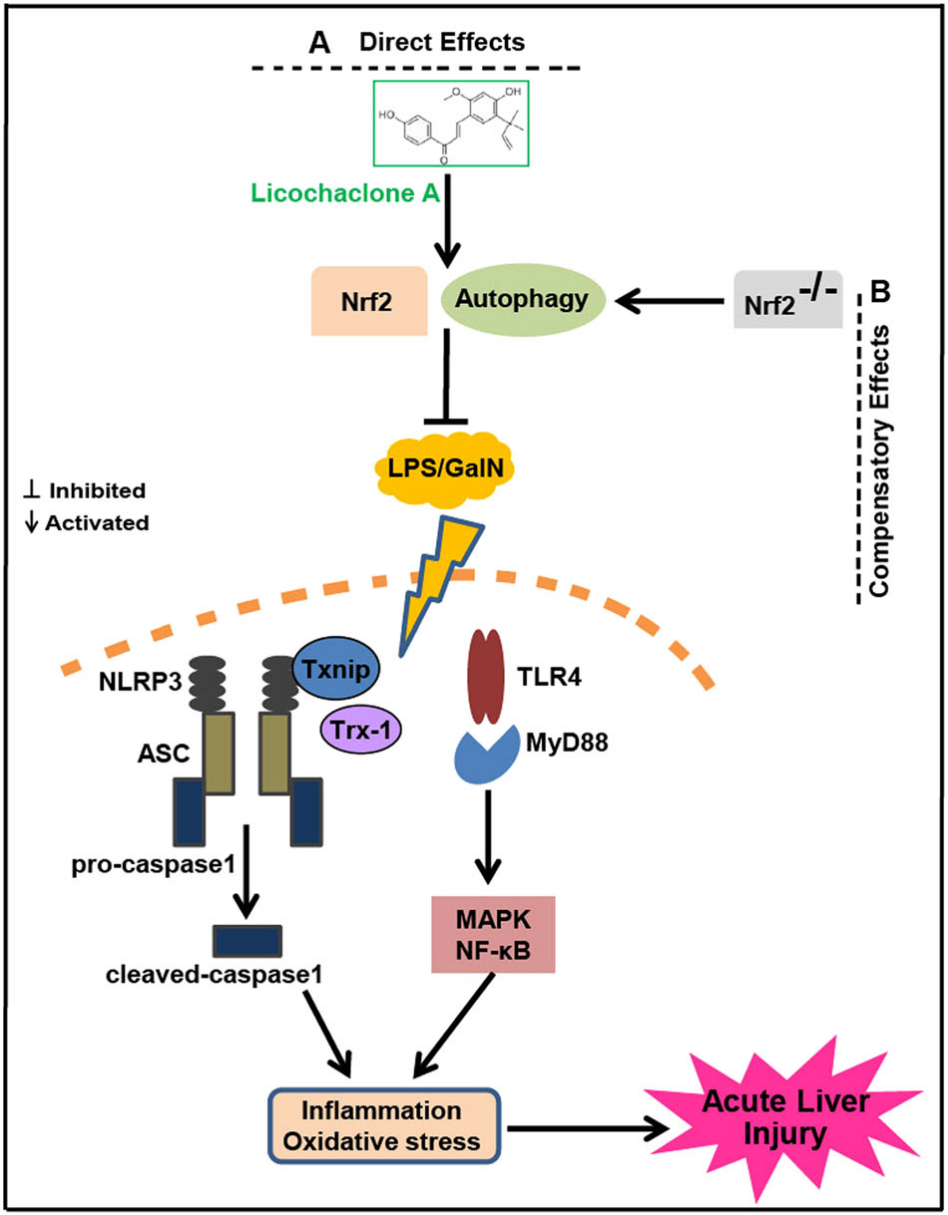
Scheme of the protective effects of Lico A on LPS/GalN-induced acute liver injury (ALI). **a** Direct effects. Lico A could induce autophagy activation and Nrf2 upregulation to alleviate inflammation and oxidative stress through the inhibition of Txnip-NLRP3 as well as TLR4-NF-κB and -MAPK signaling pathway activation, which improves LPS/GlaN-caused by ALI. **b** Compensatory effects. Knockout of Nrf2 may promote compensatory effects of autophagy activation which reduces the sensitivity of mice to LPS/GlaN; and strengthen Lico A-induced autophagy activation which enhances the ability of Lico A to protect liver injury in Nrf2^−/−^ mice

In conclusion, as presented in Fig. 10, we identified Lico A as a factor that exerts hepatoprotective activity through possible mechanisms involved in the activation of Nrf2 and induction of autophagy. Under conditions of Nrf2 absence, both LPS/GalN and Lico A may promote compensatory autophagy activation to offer benefits during liver injury. Collectively, the study provides new insights into the functional mechanism of Lico A in protecting the liver from inflammatory and oxidative stress damage during acute liver injury.

## Materials and methods

### Reagents

Licochalcone A (Lico A), purity up to 98%, was provided by the Chengdu Herbpurify CO., LTD (Chengdu, China). Dimethyl sulfoxide (DMSO), d-Galactosamine, LPS (Escherichia coli 055:B5), and inhibitors of autophagy (3-methyladenine, 3-MA) were offered by Sigma-Aldrich (St. Louis, MO, USA). Antibodies against P-AMPK/AMPK, Nrf2, HO-1, and Lamin B were offered by Abcam (Cambridge, MA, USA); and Atg7, Atg16, Beclin1, Atg12, Atg5, Atg3, LC3, NLRP3, ASC, Casapase-1, IL-1β, P-ERK/ERK, P-P38/P38, P-P65/P65, P-IκBα/IκBα, P-JNK/JNK, TFEB, P-P62, and β-actin were obtained from Cell Signaling. Additionally, ALT, AST, MDA, ROS, GSH, and SOD test kits were obtain Nanjing Jiancheng Bioengineering Institute (Nanjing, China). All other reagents were purchased from Sigma-Aldrich (St. Louis, MO, USA), if not otherwise indicated.

### Animals

Male C57BL/6 mice with Nrf2^−/−^ (Knockout) and wild-type (WT), weight 18–22 g, 6–8-week-old, were offered by The Jackson Laboratory (Bar Harbor, ME, USA) and provided by Liaoning Changsheng Technology Industrial Co., LTD (Certificate SCXK2010-0001; Liaoning, China), respectively. These animals were fed for 3 days under SPF-condition. All studies were implemented according to the International Guiding Principles for Biomedical Research Involving Animals, which was stated in the Council for the International Organizations of Medical Sciences.

### Experimental protocol

To induce acute liver injury, several protocols were carried out. First, the survival rate observation: Protocol 1, WT mice were randomly divided into five groups: Control (PBS+0.1% DMSO) group, Lico A (100 mg/kg dissolved in 0.1% DMSO) only group, LPS/GalN (30 μg/kg and 600 mg/kg dissolved in PBS) group, Lico A (50 mg/kg)+LPS/GalN group, and Lico A (100 mg/kg)+LPS/GalN group, were administered intraperitoneally. In brief, Lico A (50 or 100 mg/kg) was administered twice intraperitoneally to mice (interval of 12 h), followed by treatment with LPS (30 μg/kg) and GalN (600 mg/kg), which is abbreviated as LPS/GalN. The survival rates of mice were observed for 24 h after LPS/GalN challenge. Protocol 2, WT or Nrf2^−/−^ mice were respectively separated into four groups: Control group, Lico A (100 mg/kg) only group, LPS/GalN (30 μg/kg and 600 mg/kg) group and Lico A (100 mg/kg)+LPS/GalN groups were administered intraperitoneally. The survival rates of mice were observed for 24 h after LPS/GalN challenge. Protocol 3, WT mice were randomly divided into six groups: Control group, Lico A (100 mg/kg) only group, LPS/GalN (10 μg/kg and 700 mg/kg) group, 3-MA (20 mg/kg)+LPS/GalN group, Lico A (100 mg/kg)+LPS/GalN group, and 3-MA+Lico A+LPS/GalN, were administered intraperitoneally. In brief, mice were intraperitoneally injected 3-MA (20 mg/kg) for 2 h, and then Lico A (100 mg/kg) with mice for twice (interval for 12 h), followed by subjected to LPS (10 μg/kg)/GalN (700 mg/kg) according to previous described^42^. Second, the mechanism analysis: the mice subjected to protocol 1 or 2 were administered. Subsequently, liver tissues and serum were gathered after LPS/GalN for 3 h and 6 h, which were used for biochemical indexes assay, hematoxylin and eosin (H & E) staining, ELISA or western blotting assay.

### Histopathological evaluation

Fresh liver tissues were gathered, fixed instantly with formalin, and then embedded in paraffin. Lastly, the liver tissues were cut into a thickness of 5 μm sections, which were stained with hematoxylin-eosin (H&E) staining to assess the liver pathological changes using a light microscopy. The histological changes were evaluated by a point-counting method for severities of hepatic injury using an ordinal scale in accordance with the methods as previous described^49^. The stained sections were graded as a four-point scale from 1 to 4 as follows: 1, 2, 3, and 4 represent no damage, mild damage, moderate damage, and severe damage, respectively.

### Measurement of ALT, AST, MDA, ROS, SOD, and GSH levels

All mice were killed at 3 h or 6 h after L/D treatment, liver and blood were collected for biochemical analysis. ALT and AST levels in serum and liver were measured using the corresponding detection kits in accordance with the manufacturer’s instructions. In addition, mice liver tissues were homogenized and dissolved in extraction buffer to analyze the MDA, ROS, SOD, and GSH levels according to the manufacturer’s instructions. All results were normalized by the total protein concentration in each sample.

### ELISA assay

Blood was obtained from each sample in vivo, centrifuged, collected serum for measurement of the TNF-α, IL-6, and IL-1β secretion using an enzyme-linked immunosorbent assay (ELISA) kit as the manufacturer’s instructions (BioLegend, Inc., CA, USA), respectively. The optical density from each well was detected at 450 nm.

### Western blot analysis

Liver tissues were collected 3 h or 6 h after L/D challenge. Total protein was extracted from the liver tissues using a protein extract kit according to the manufacturer’s protocol. Protein concentrations were tested by the BCA method. Equal amounts of proteins (20 μg) were separated by a 10% SDS-polyacrylamide gel and transferred onto a polyvinylidene difluoride (PVDF) membrane. The membrane was blocked with 5% (w/v) nonfat milk for 2 h. Then, the membrane was incubated with primary antibody and secondary antibody. Finally, the membranes were visualized by the ECL Western blotting detection system in accordance with the manufacturer’s instruction and band intensities were quantified using Image J gel analysis software. All experiments were performed in triplicate.

### Statistical analysis

All data referenced above were expressed as the means±SEM and analyzed using SPSS19.0 (IBM). Comparisons between experimental groups were conducted using one-way ANOVA, whereas multiple comparisons were made using the LSD method. Statistical significance was defined as *p* < 0.05 or *p* < 0.01.
